# Oral complications associated with trigeminal trophic syndrome: A case report

**DOI:** 10.1111/scd.13085

**Published:** 2024-11-14

**Authors:** Temitope Omolehinwa, Andres Davila, Roopali Mahajan, Thomas P. Sollecito, Eric T. Stoopler, Roopali Kulkarni

**Affiliations:** ^1^ Department of Oral Medicine University of Pennsylvania School of Dental Medicine Philadelphia USA; ^2^ Department of Applied Dental Medicine Section of Diagnostic Sciences Southern Illinois University School of Dental Medicine Alton USA; ^3^ Value Dental of Arlington Arlington USA

**Keywords:** nasal ala, oral trauma, trigeminal ganglion, trigeminal trophic syndrome, ulceration

## Abstract

**Introduction:**

Destruction of the trigeminal (Gasserian) ganglion or peripheral damage to the trigeminal nerve may lead to trigeminal trophic syndrome (TTS), a rare condition characterized by self‐inflicted trauma. To date, under 200 cases of TTS are documented in medical literature, with only sparse studies reporting on oral complications secondary to this condition.

**Case report:**

The following report presents a well‐documented case of oral complications associated with TTS in an 83‐year‐old Caucasian female presenting with right‐sided lesions on her nasal ala, scalp, buccal mucosa, and tongue, secondary to self‐inflicted injury following their second microvascular decompression for surgical management of trigeminal neuralgia.

**Discussion/conclusion:**

The paucity of literature on oral complications associated with TTS supports the need for understanding guiding management modalities of such lesions for clinical practice. To the authors’ knowledge, this is the first manuscript detailing present and potential intraoral complications associated with TTS alongside treatment modalities for the practicing clinician.

## 1 INTRODUCTION

The trigeminal system comprises the largest cranial nerve in the human body and provides sensory innervation to the facial region.^1^ Trigeminal trophic syndrome (TTS) is a rare condition characterized by self‐inflicted trauma, resulting from the destruction of the trigeminal ganglion or peripheral damage to the trigeminal nerve (TN). ^2^, ^3^ This leads to facial dysesthesias along the TN pathway and subsequent development of painless, persistent facial trauma and self‐mutilation within the affected TN dermatomes. To date, under 200 cases of TTS are documented in the medical literature, with only sparse studies reporting on oral manifestations secondary to this condition.^3^, ^4^, ^5^ Herein, we present a case of TTS following microvascular decompression for trigeminal neuralgia, highlighting intraoral complications associated with TTS. To the authors’ knowledge, this is the first manuscript detailing present and potential intraoral manifestations secondary to TTS alongside treatment modalities for the practicing clinician.

### 1.1 Case report

An 83‐year‐old female presented with a one‐year history of right‐sided lesions on her nasal ala, scalp, buccal mucosa, and tongue, secondary to self‐injury. Eleven years prior, following her second microvascular decompression for trigeminal neuralgia, the patient began to experience right‐sided hypoesthesia in both the extraoral and intraoral regions, along with pruritus and a “crawling sensation” predominantly on the right nasal ala. The symptoms affected all three divisions of the trigeminal nerve and were attributed to an iatrogenic injury of the trigeminal ganglion.

Following thorough evaluation, a neurologist confirmed a diagnosis of right‐sided TTS and advised the patient to reduce trauma by using protective dressings. The patient subsequently sought care from a dermatologist, who prescribed topical pimecrolimus 1% for the extraoral lesions. However, no significant improvement was observed with this intervention. Pertinent negatives in the patient's medical history, included infections, neoplasms, and Morgellons disease. The patient's medical history included atrial fibrillation, anxiety, Meniere's disease, and right lower extremity necrotizing fasciitis status‐post below‐the‐knee amputation, and management of related pain. Her current medication regimen comprised duloxetine, amitriptyline, and gabapentin.

**FIGURE 1:**
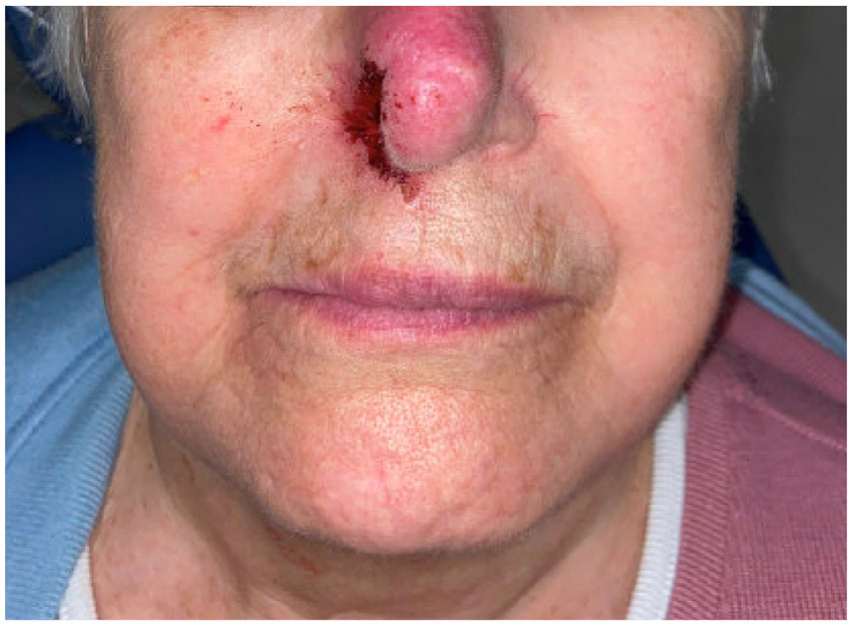
Full face frontal view—evident asymmetry of the nasolabial folds with erythema of the nose and right‐sided scabbing of the nasal ala.

**FIGURE 2:**
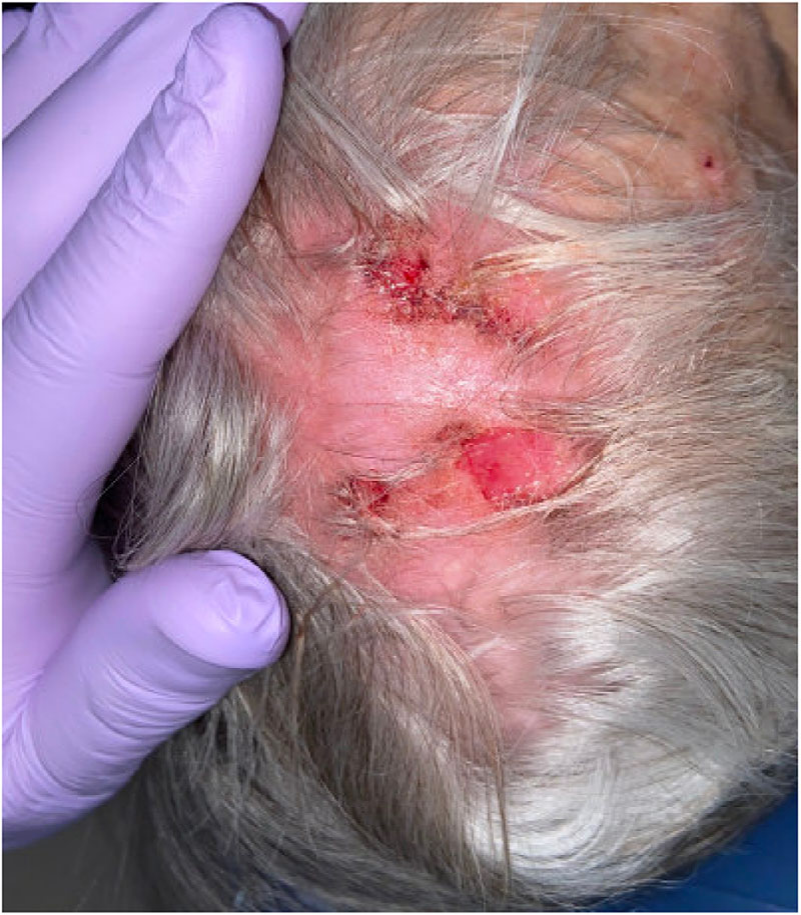
Coronal view—right‐sided erosions on the scalp.

**FIGURE 3:**
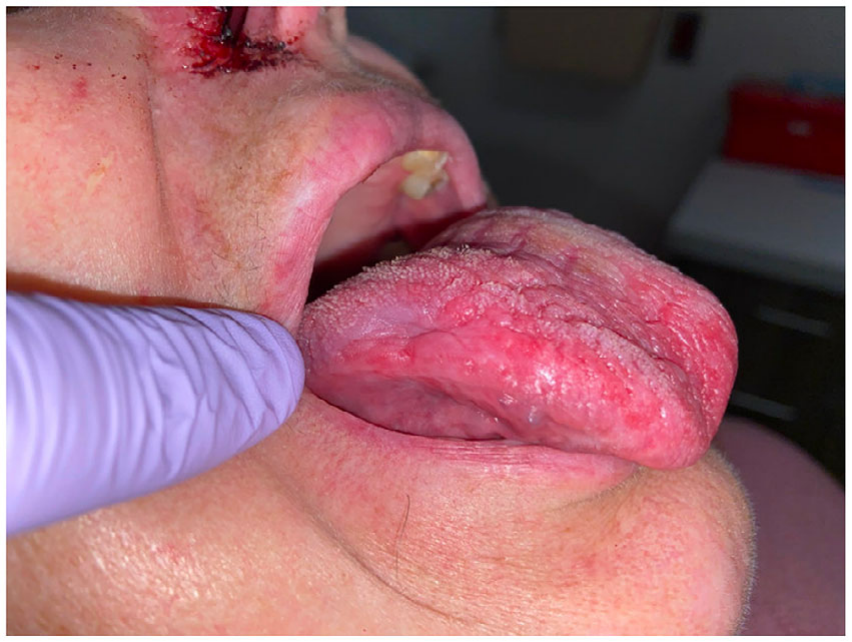
Intraoral view of the right lateral border of the tongue—coated, fissured, and erythematous tongue with a non‐indurated 3 × 2  mm healing ulceration on the right lateral border.

A comprehensive review of systems revealed no additional significant findings. On cranial nerve examination, the patient exhibited right‐sided extraoral and intraoral hypoesthesia in all divisions of the trigeminal nerve. No motor deficits were observed during the examination. Physical examination revealed asymmetric nasolabial folds (Figure 1), crescentic ulceration on the right nasal ala, and right‐sided scalp erosions (Figure 2). An intraoral examination revealed a firm, normal‐colored, ulcerated 5 × 6  mm nodule in close proximity to the right oral commissure. Additionally, the tongue displayed a coated and fissured appearance with a non‐indurated 3 × 2  mm healing ulceration detected on the right lateral border (Figure 3). The patient also reported a dry mouth sensation. Notably, both the nodular lesion and healing ulceration were located in regions associated with reported dysesthesia.

The assessment was consistent with right‐sided TTS based on the medical history and clinical presentation, known intraoral trauma secondary to TTS, and medication‐induced xerostomia. The patient was referred to behavioral health for behavioral modification, otorhinolaryngology for extra‐oral wound care, and planned excisional biopsy of the nodule. The patient was recommended to use over‐the‐counter sialagogues and was prescribed high‐fluoride toothpaste for caries‐risk reduction given their medication‐induced xerostomia. The patient did not return to office for excisional biopsy and remained under the care of otorhinolaryngology and behavioral health. Cutaneous biopsies were not recommended at the time given the traumatic nature of the lesions and subsequent focus on extra‐oral wound care.

## 2 DISCUSSION

First appearing in the early 20th‐century medical literature, TTS was observed in patients who underwent trigeminal rhizotomy to manage trigeminal neuralgia.^6^, ^7^ TTS combines intractable neuropathic pruritus and extensive cutaneous deafferentation, resulting in painless self‐mutilation.^8^ The most common causes of TTS include stroke followed by trigeminal ablation, surgical complication, herpes zoster, trauma, and meningioma complication, respectively.^2^ Additionally, TTS has been reported following alcohol injection of the trigeminal nerve and coagulation of the Gasserian ganglion for treatment in trigeminal neuralgia.^8^ Nearly all cases of TSS are unilateral, though one suspected case discusses bilateral TSS, which may indicate the possibility of TSS following bilateral surgical procedures including oncologic resection or facial injury repair.^9^

TTS has been known to afflict a wide range of age groups, from 14 months to 94 years (mean = 57 years), with female predilection of slightly over 2:1.^8^ The latency period from initial injury to symptom onset ranges from 0 days to 30 years (mean = 5 years).^2^ The classic triad of clinical symptomatology for TTS is a history of trigeminal sensory impairment, altered facial sensations, and a crescent‐shaped ulceration of the nasal ala.^2^ Patient‐reported sensations and symptoms may include anesthesia, pruritus, tickling, burning, “crawling sensation,” nasal blockage, or drainage in the nasopharynx.^10^ Facial trauma is self‐inflicted due to habitual self‐manipulation in response to reported sensations. Resultant ulcerations may be localized or include multiple areas of involvement. The most common location is the nasal ala, with less common sites including the scalp and eyelid.^2^

TTS is a complex entity requiring a thorough intake of the history of the present illness, physical assessment for signs of involvement, and the need to rule out other similarly presenting conditions. The differential diagnoses of TTS include conditions associated with facial ulcerations, such as factitial injuries, physical abuse, methamphetamine excoriations, gangrene/noma, infections, vasculitis, granulomatous disease, and cutaneous neoplasms (Table 1). Additionally, liver disease, mastocytosis, eczema, lichen planus, and psoriasis are associated with pruritus and may need to be considered in diagnostic workup.^11^ Given TTS is a diagnosis of exclusion, a tissue biopsy and blood investigations may be recommended to rule out other causative factors. Blood orders may include those associated with monitoring infection or inflammation in autoimmune diseases.^12^, ^13^ Timely diagnosis is critical to minimize the extent of soft tissue injury.

**TABLE 1:** Differential diagnosis of trigeminal trophic syndrome.

Diagnosis	Differentiating characteristics
Infection (e.g. Herpes, Syphilis, Leprosy, Tuberculosis, Mycobacteria, Deep Fungal Infection, Leishmaniasis)^3^, ^20^	Prodromal symptoms of fever, fatigue, headache, malaise and photophobia. Unilateral dermatomal distribution initiated as maculopapular rash on an erythematous base, evolving into vesicular‐pustular appearance. Presence of very sensitive skin. Presence of multinucleated giant cells on Tzanck smear or positive PCR. Positive bacterial and/or fungal tests.
Excoriation Disorders and Methamphetamine Overuse^21^	Chronic skin picking or scratching associated with methamphetamine overuse. May pick at skin on face, hands, arms, legs.
Factitial Injuries^22^	Typically characterized by underlying psychological condition. May be associated with illness deception, malingering, somatoform disorders, or dissociative disorder.
Physical Abuse^23^	Defined by intentional bodily injury from one party to another. Can be characterized by bruising, erosions, abrasions. Not necessarily unilateral or present in the orofacial region alone.
Noma (orofacial gangrene)^24^	Characterized by orofacial gangrene. Typically seen in malnourished children. Mainly observed in tropical countries.
Systemic Vasculitis (i.e., Granulomatosis with Polyangiitis)^3^	Biopsy exhibits periadventitial vasculitis. Presence of multisystem involvement including renal (glomerulonephritis) and pulmonary (multiple parenchymal lesions). Associations with systemic conditions such as Crohn's disease and sarcoidosis. Positive laboratory assays for immune complexes, antinuclear factor, antineutrophil cytoplasmic antibody, and perinuclear antineutrophil cytoplasmic antibody.
Pyoderma Gangrenosum^3^, ^25^	Rare location on the face (common on lower extremity). Painful lesions. Associated with underlying systemic conditions such as rheumatoid arthritis, inflammatory bowel disease (IBD), hematological malignancies or monoclonal gammopathies, chronic infection and inflammation. Cutaneous biopsy to exclude other causes of cutaneous ulceration. Laboratory investigations: Complete blood count, erythrocyte sediment rate, C‐reactive protein, liver and renal function tests, protein electrophoresis, urinary Bence Jones protein, hepatitis screen, a vasculitic screen, coagulation screen to assess the thrombotic causes of ulceration. A fecal calprotectin is advised in case of suspected IBD. Chest x‐ray and computed tomography are indicated if there is a suspicion of underlying malignancy.
Dermatitis Artefacta^3^, ^26^	History of underlying stress or psychological disorder. No sensory loss. Patients deny self‐inflicted injury. Histopathology: epidermal necrosis, subepidermal/intraepidermal blistering, foreign body granulomas which are usually nonspecific, the presence of ≥5 multinucleated keratinocytes (characteristic finding).
Neoplasms (e.g. Squamous Cell Carcinoma, Basal Cell Carcinoma, Lymphoma, Sarcoma)^3^	Histopathology: atypical/malignant/neoplastic cells.

Adapted from Khan AU, Khachemoune A. Trigeminal trophic syndrome: an updated review.^3^

To date, there is no standard of care for the management of TTS. Current treatment enforces a triad of treatment modalities: (1) behavioral management to address habitual skin manipulation, (2) tissue debridement and maintenance to prevent infections, and (3) pharmacologic management for neuropathic symptomatology. Surgical considerations have been reported with varying success; however, improvement in behavioral tendencies is first recommended to avoid severe soft tissue defects.^2^, ^3^ Surgical options for extensive injury may include nerve, skin, or cartilage grafting, including crossface grafting, and composite tissue transplantation.^14^ Diagnosis and co‐management of the condition may bring together specialists in neurology, dermatology, otorhinolaryngology, oral and maxillofacial surgery, plastic surgery, and infectious disease.

Recent advances in therapeutic approaches and methodologies for TTS hold promising prospects. Wang and Li exhibited a potential treatment option for TTS using botulinum toxin type A in combination with pulsed radiofrequency to the semilunar ganglion of the trigeminal nerve for TTS, relieving the itch of the patient with significant subsequent healing of associated ulceration.^15^ By utilizing a thermoplastic mask sutured in place overlying chronic ulcerations associated with TTS, Pisano et al. observed enhanced re‐epithelialization and improved wound healing over a 6‐week period.^16^ These cases highlight the potential of innovative approaches to wound management that could enhance healing outcomes in patients with TTS. In rare cases, patients may even develop secondary infection or facial cellulitis, posing an immediate medical emergency.^12^ This also exemplifies the importance of timely diagnosis and management.

In the present case, our patient presented with right‐sided self‐inflicted extra‐ and intra‐oral lesions in the v1, v2, and v3 dermatomes of the TN. Sensory and motor innervation of the face play an essential role in tissue protection.^17^ The buccal and lingual nerves are branches of the mandibular division of the trigeminal nerve and transmit sensory information. The buccal nerve innervates the buccal mucosa and the tissue of the oral commissure, and the lingual nerve innervates the anterior two‐thirds of the tongue. Trigeminal nerve injury can result in various clinical manifestations such as buccal and lingual branch involvement, leading to symptoms of dysesthesia, anesthesia, or hypoesthesia. Such sensory deficits may potentially increase the likelihood of self‐inflicted injuries.^18^ In our patient, an assessment of the cranial nerve function revealed right‐sided intraoral hypoesthesia, which corresponded to the presence of traumatic lesions in the same area. This observation underscores the significance of neurosensory examination in the diagnosis and management of oral and facial pain and headache conditions.

In light of the patient's neuropathy/TTS, oral healthcare professionals should acquaint themselves with the various oral mucosal lesions that may arise as a consequence of trauma (Table 2). Furthermore, it is imperative to recognize that medication‐induced xerostomia and hyposalivation are common adverse effects associated with the pharmacotherapy employed in the neuropathic management of TTS, including antidepressants and anticonvulsants, as noted in the literature.^19^ When managing xerostomia, a conservative approach should be considered by the clinician, prioritizing interventions such as salivary gland massage or over‐the‐counter sialagogues when necessary, along with the use of prescription‐strength fluoride toothpaste to mitigate the risk of dental caries. Prescription sialagogues, such as pilocarpine and cevimeline, have been found to be effective in managing xerostomia by stimulating salivary flow and alleviating the symptoms associated with reduced salivary production, including oral discomfort, difficulty in speaking and swallowing, and an increased risk of dental caries. They are particularly useful when conservative therapies are inadequate in addressing the patient's dry mouth symptoms. However, individualized treatment plans should be developed after considering potential side effects and contraindications of prescription sialagogues and discussing alternative medications for currently prescribed medications with an increased risk of anti‐cholinergic properties. It is important to acknowledge that due to the patient being lost to follow‐up, the intended therapeutic strategies outlined in our report could not be fully implemented, thus imposing a limitation on our findings. Nevertheless, the proposed treatment recommendations for intraoral trauma (Table 2) have gained widespread adoption in current clinical practice.

**TABLE 2:** Summary of potential intraoral traumatic lesions in the setting of trigeminal trophic syndrome with management modalities.

Condition	Etiology	Clinical manifestation	Diagnosis	Management
Frictional Keratosis (e.g. Linea Alba, Benign Alveolar Ridge Keratosis, Morsicatio Buccarum, Morsicatio Labiorum, Morsicatio Linguarum)^27^	Associated with mechanical irritation (e.g. biting, brushing, denture trauma, teeth rubbing against mucosa)	Asymptomatic, poorly defined, unilateral or bilateral, white plaques or papules May present on the buccal mucosa, labial mucosa, retromolar area, edentulous alveolar ridge, and/or lateral tongue	Clinical presentation Adjunct biopsy may be necessitated in atypical cases	Elimination of traumatic source with close follow‐up; re‐evaluate in two weeks If lesion persists, biopsy is warranted to rule out dysplasia/malignancy
Traumatic Ulcers^28^	Associated with acute or chronic trauma	Acute trauma: symptomatic, yellow‐tan base with erythematous borders Chronic trauma: asymptomatic, yellow‐tan center with raised, white, indurated, rolled, margins; Persist >two weeks, Most common location is the border of the tongue.	Clinical presentation and history Adjunct biopsy necessitated for oral ulcer persisting >two weeks despite removal of suspected initiating factors	Elimination of traumatic source with close follow‐up; re‐evaluate in two weeks Pharmacologic management: may use topical corticosteroids (e.g. fluocinonide or clobetasol gel) Palliative care: may use topical anesthetic (e.g. lidocaine gel) Adjunct biopsy necessitated for oral ulcer persisting >two weeks despite removal of suspected initiating factors to rule out dysplasia/malignancy/manifestation of systemic disease/deep fungal infection
Irritation Fibroma^27^	Associated with mechanical trauma and chronic irritation	Asymptomatic, firm, smooth, sessile, normal‐colored (similar to surrounding mucosa) nodular lesion May present on the buccal mucosa, labial mucosa, and lateral tongue	Clinical presentation and histopathological analysis	Excisional biopsy for symptomatic lesions Low recurrence rate
Mucocele^29^	Associated with mechanical trauma and chronic irritation (e.g. lip biting, lip sucking) resulting in damage to minor salivary glands or ducts.	Asymptomatic, fluctuant, translucent, mobile, pseudo‐cystic lesion May rupture revealing mucous exudate Most common location lower labial mucosae	Clinical presentation, location, history of trauma	May resolve without treatment Surgical excision with removal of surrounding minor salivary glands commonplace Chance of recurrence
Thermogenic^30^	Associated with transfer of latent heat upon contact with extreme temperatures	Immediate burn of mucosae upon contact evolving into vesicular or ulcerative lesions with hyperemic border	Clinical presentation and history of trauma	Pharmacologic care and close follow‐up Pharmacologic management: may use topical corticosteroids (e.g. fluocinonide or clobetasol gel) or Palliative care: may use topical anesthetic (e.g. lidocaine gel) Temporarily avoid ingestion of substance which may irritate existing lesion (e.g. spicy foods, acidic drinks)

## 3 CONCLUSION

The lack of literature addressing interventions for oral complications associated with TTS underscores the significance of generating discourse and publications that offer guidance on treatment strategies for clinical application. This case report contributes to the limited body of literature on TTS and sheds light on potential traumatic oral complications that may arise as a result of this rare condition. It is recommended that future studies and reports place emphasis on intraoral complications secondary to this uncommon disorder to facilitate better understanding and management of this challenging condition.

## CONFLICT OF INTEREST STATEMENT

The authors declare no conflicts of interest.

Work from this case series was accepted as an abstract and presented as a poster at the Academy of General Dentistry 2022 Meeting, the Dermatology Education Foundation's Nurse Practitioner and Physician's Assistant Continuing Medical Education Derm 2022 Conference, and the National Organization for Rare Disorders Breakthrough Summit 2022.
